# Phenotype of ASDs Associated With 4p16 Risk Locus and Novel Genome-Wide Associations of ASD Patients in the Finnish Population

**DOI:** 10.1161/CIRCGEN.123.004070

**Published:** 2023-08-14

**Authors:** Valtteri Muroke, Mikko Jalanko, Sanni Ruotsalainen, Markus Perola, Emmi Helle, Juha Sinisalo

**Affiliations:** Department of Cardiology, Helsinki University Hospital (V.M., M.J., J.S.), University of Helsinki, Finland.; Institute for Molecular Medicine Finland (FIMM), HiLIFE (S.R.), University of Helsinki, Finland.; Stem Cells and Metabolism Research Program, Faculty of Medicine (E.H.), University of Helsinki, Finland.; New Children’s Hospital, Pediatric Research Center, Helsinki University Hospital and University of Helsinki, Finland (E.H.).; Finnish Institute for Health and Welfare, Helsinki, Finland (M.P.).

**Keywords:** genetic loci, genome, heart defects, congenital, heart septal defect, atrial, mutation

Atrial septal defect (ASD) is a common congenital heart defect characterized by communication between the left and right atria. Mutations in genes essential to cardiac septation, most notably *NKX2-5*, *GATA4*, and *TBX5*, are associated with ASD formation.^1^ First-degree relatives of isolated patients with ASD have an increased risk for ASD compared with controls.^2^

There is substantial heritability among sporadic ASD cases, where the genetic cause is unknown. This study aimed to find new susceptibility loci for ASD in a genome-wide association study. Furthermore, we evaluated whether the identified variants are related to the echocardiographic anatomy of the defect.

We identified all patients diagnosed with isolated ASD from the FinnGen database. In addition, patients with procedure codes for surgical or percutaneous ASD closure or ASD as a cause of death were included. Patients with diagnoses of other congenital heart diseases were excluded. In total, 1174 ASD cases (719 female patients) were included. The control population consisted of 407 475 individuals without any congenital malformations of the circulatory system. A genome-wide association study used the regular FinnGen pipeline, and *P*<5e-8 was considered statistically significant. R software was used to generate the Manhattan plot. Furthermore, expression quantitative trait loci analyses were conducted using GTExPortal.

Detailed chart review was performed for those FinnGen patients with ASD included in Helsinki Biobank, one of the biobanks submitting samples to FinnGen. In total, 178 patients with ASD and genome data were identified in the biobank. Of those, 14 were excluded (7 had patent foramen ovale, 2 had other congenital heart defect, and 5 had no data available), resulting in 164 patients.

Patients and control subjects in FinnGen provided informed consent for biobank research, based on the Finnish Biobank Act. Alternatively, separate research cohorts, collected before the Finnish Biobank Act came into effect (in September 2013) and start of FinnGen (August 2017), were collected based on the study-specific consents and later transferred to the Finnish biobanks after approval by the Finnish Medicines Agency, the National Supervisory Authority for Welfare and Health. Recruitment protocols followed the biobank protocols approved by the Finnish Medicines Agency. The Coordinating Ethics Committee of the Hospital District of Helsinki and Uusimaa (HUS) statement number for the FinnGen study is Nr HUS/990/2017. The FinnGen study is approved by the Finnish Institute for Health and Welfare (permit numbers: THL/2031/6.02.00/2017, THL/1101/5.05.00/2017, THL/341/6.02.00/2018, THL/2222/6.02.00/2018, THL/283/6.02.00/2019, THL/1721/5.05.00/2019, and THL/1524/5.05.00/2020), Digital and population data service agency (permit numbers: VRK43431/2017-3, VRK/6909/2018-3, and VRK/4415/2019-3), the Social Insurance Institution (permit numbers: KELA 58/522/2017, KELA 131/522/2018, KELA 70/522/2019, KELA 98/522/2019, KELA 134/522/2019, KELA 138/522/2019, KELA 2/522/2020, and KELA 16/522/2020), Findata permit numbers THL/2364/14.02/2020, THL/4055/14.06.00/2020, THL/3433/14.06.00/2020, THL/4432/14.06/2020, THL/5189/14.06/2020, THL/5894/14.06.00/2020, THL/6619/14.06.00/2020, THL/209/14.06.00/2021, THL/688/14.06.00/2021, THL/1284/14.06.00/2021, THL/1965/14.06.00/2021, and THL/5546/14.02.00/2020), and Statistics Finland (permit numbers: TK-53-1041-17 and TK/143/07.03.00/2020 [earlier TK-53-90-20]). The Biobank Access Decisions for FinnGen samples and data used in FinnGen Data Freeze 7 include the following: THL Biobank BB2017_55, BB2017_111, BB2018_19, BB_2018_34, BB_2018_67, BB2018_71, BB2019_7, BB2019_8, BB2019_26, BB2020_1, Finnish Red Cross Blood Service Biobank 7.12.2017, Helsinki Biobank HUS/359/2017, Auria Biobank AB17-5154 and amendment 1 (August 17, 2020), Biobank Borealis of Northern Finland_2017_1013, Biobank of Eastern Finland 1186/2018 and amendment 22 §/2020, Finnish Clinical Biobank Tampere MH0004 and amendments (21.02.2020 and 06.10.2020), Central Finland Biobank 1-2017, and Terveystalo Biobank STB 2018001.

The ethics committee of the Hospital District of HUS approved this study on April 27, 2022 (HUS/11721/2022). The study was conducted following the Declaration of Helsinki, and patient consent was not needed for register-based research. Data are available from the authors on reasonable request and with permission from FinnGen and Helsinki Biobank.

Three genome-wide significant associations were identified: rs4689912 (*P*=1.1e-12; odds ratio, 1.37 [95% CI, 1.26–1.50], intronic region of STX18-AS1), rs1207492464 (*P*=4.5e-11; odds ratio, 7.94 [95% CI, 4.56–13.83], intronic region of BAHCC1), and rs1429652525 (*P*=2.0e-8; odds ratio, 5.48 [95% CI, 3.06–9.82], downstream AC091576.1, upstream MC4R; Figure). Allele frequencies were 0.32 and 0.25 (rs4689912), 0.0076 and 0.00087 (rs1207492464), and 0.0080 and 0.0019 (rs1429652525) in patients with ASD and controls, respectively. The inflation factor lambda 0.5 was 1.001. For rarer variants, the *P*-values were 0.097 for BAHCC1 and 0.32 for AC091576.1 in burden analysis (minor allele frequency <0.1%).

**Figure.**
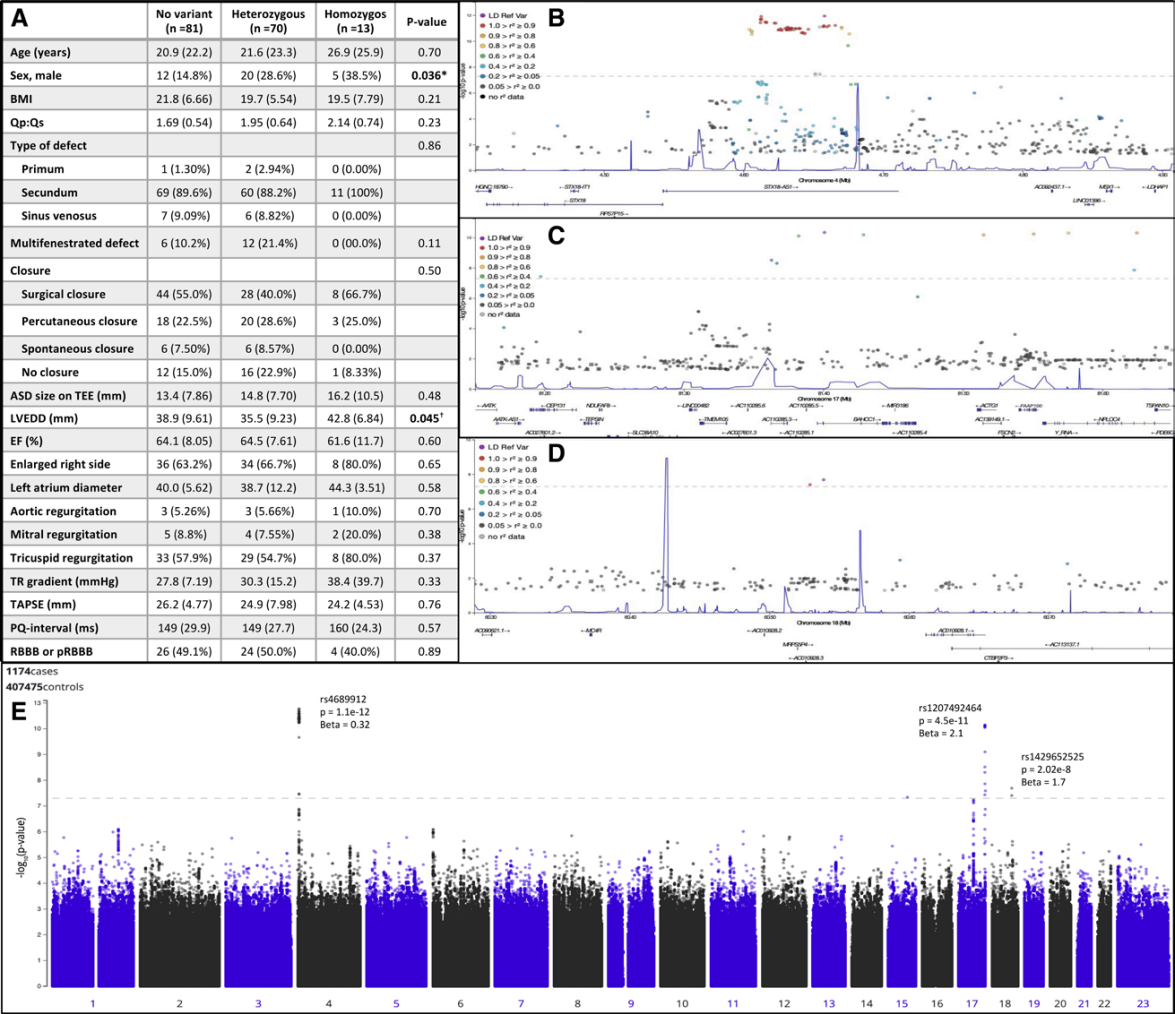
**Differences in atrial septal defect (ASD) characteristics in patients with and without intronic STX-18-AS1 variant and regions associated with ASD.** Continuous variables were compared using ANOVA, and categorical variables were compared using Fisher exact test (**A**). Locus zoom-plot of the regions associated with ASD in chromosome 4 (**B**), 17 (**C**), and 18 (**D**). Values are represented as mean (SD) or the number of patients (%). Age refers to the age at the time of closure or diagnosis if the defect is unclosed. *The difference was only significant between heterozygous patients and patients with no variant (*P*=0.047), but not between other groups. †The difference was only significant between heterozygous and homozygous patients (*P*=0.015) but not between other groups. BMI indicates body mass index; EF, ejection fraction; LVEDD, left ventricular end-diastolic diameter; TAPSE, tricuspid annular plane systolic excursion; TEE, transesophageal echocardiography; and TR, tricuspid regurgitation.

In the rs4689912 locus, there was no significant association with any nearby single nucleotide polymorphisms to the expression of nearby genes in heart tissue in the expression quantitative trait loci analyses, and rs1207492464 and rs142965252 were not found in the GTExPortal. In the FinnGen PheWAS data for other cardiac phenotypes, the lead single nucleotide polymorphisms rs4689912 and rs1207492464 were associated with congenital heart defects in general, and rs1207492464 was also associated with unspecified cardiomyopathy.

Of the 164 biobank subjects with ASD and FinnGen data, 13 were homozygous for the STX18-AS1 intronic variant, 70 were heterozygous, and 81 did not have the variant. Patients with ASD with the STX-18-AS1 intronic variant were more often male (Figure). According to the retrospective chart review, there was no significant difference in the defect type (primum, secundum versus venosus; *P*=0.86), defect size (*P*=0.25), shunt size (*P*=0.78), or defect count (single versus multifenestrated, *P*=0.11; Figure). All patients who were homozygous for the intronic STX18-AS1 variant had a secundum-type defect, and none had spontaneous closure.

Two patients in the biobank population had the intronic BAHCC1 variant rs1207492464, which are not present in other populations in gnomAD than Finnish. Also, rs1429652525 (nearest gene AC091576.1) was Finnish enriched (AF 0.0016 Finnish and 0.000007 in other gnomAD populations) and was found in 2 patients in the biobank population.

We identified 3 loci associated with ASD, 2 of which were Finnish enriched and novel. The third replicated the finding of the association of the 4p16 locus and ASD shown in earlier genome-wide association studies.^3,4^ STX-18-AS1 is known to act as a regulator for NKX2-5, a key cardiac transcriptional factor.^5^ Furthermore, we retrospectively reviewed the clinical data and the echocardiographic anatomy of ASD from electronic health records. No significant difference was found in the anatomy of the ASD between patients with and without the intronic STX-18-AC1 variants. All patients homozygous for the variant had secundum-type defects, of which none had spontaneous closure. These findings are limited due to the small sample size.

The 2 novel ASD risk loci rs1207492464 and rs1429652525 were Finnish enriched with relatively large effect sizes. These loci have not been associated with cardiac defects before.

In conclusion, intronic STX18-AS1 and BAHCC1 variants and locus rs1429652525 were associated with ASD. STX-18-AS-1 variants did not have a significant effect on the ASD phenotype.

## ARTICLE INFORMATION

### Acknowledgments

The authors want to acknowledge the participants and investigators of FinnGen study. The FinnGen project is funded by 2 grants from Business Finland (Helsinki and Uusimaa [HUS] 4685/31/2016 and UH 4386/31/2016) and the following industry partners: AbbVie Inc., AstraZeneca UK Ltd, Biogen MA Inc., Bristol Myers Squibb (and Celgene Corporation & Celgene International II Sàrl), Genentech Inc., Merck Sharp & Dohme LCC, Pfizer Inc., GlaxoSmithKline Intellectual Property Development Ltd., Sanofi US Services Inc., Maze Therapeutics Inc., Janssen Biotech Inc, Novartis AG, and Boehringer Ingelheim International GmbH. Following biobanks are acknowledged for delivering biobank samples to FinnGen: Auria Biobank (www.auria.fi/biopankki), THL Biobank (www.thl.fi/biobank), Helsinki Biobank (www.helsinginbiopankki.fi), Biobank Borealis of Northern Finland (https://www.ppshp.fi/Tutkimus-ja-opetus/Biopankki/Pages/Biobank-Borealis-briefly-in-English.aspx), Finnish Clinical Biobank Tampere (www.tays.fi/en-US/Research_and_development/Finnish_Clinical_Biobank_Tampere), Biobank of Eastern Finland (www.ita-suomenbiopankki.fi/en), Central Finland Biobank (www.ksshp.fi/fi-FI/Potilaalle/Biopankki), Finnish Red Cross Blood Service Biobank (www.veripalvelu.fi/verenluovutus/biopankkitoiminta), Terveystalo Biobank (www.terveystalo.com/fi/Yritystietoa/Terveystalo-Biopankki/Biopankki/), and Arctic Biobank (https://www.oulu.fi/en/university/faculties-and-units/faculty-medicine/northern-finland-birth-cohorts-and-arctic-biobank). All Finnish Biobanks are members of The Biobanking and BioMolecular Resources Research Infrastructure (BBMRI) (www.bbmri.fi). Finnish Biobank Cooperative-(FINBB) (https://finbb.fi/) is the coordinator of BBMRI-European Research Infrastructure Consortium operations in Finland. The Finnish biobank data can be accessed through the Fingenious services (https://site.fingenious.fi/en/) managed by FINBB.

### Sources of Funding

This work was supported by Emil Aaltonen Foundation, Päivikki and Sakari Sohlberg foundation, and The Foundation for Pediatric Research.

### Disclosures

None.
